# Data on the distribution of physical activities in the Shenzhen greenway network with volunteered geographic information

**DOI:** 10.1016/j.dib.2016.05.006

**Published:** 2016-05-12

**Authors:** Kun Liu, Kin Wai Michael Siu, Yong Xi Gong, Yuan Gao, Dan Lu

**Affiliations:** a The Hong Kong Polytechnic University, School of Design, Hong Kong; b Harbin Institute of Technology, Shenzhen Graduate School, Shenzhen, China

**Keywords:** Physical activity, Volunteered geographic information, Shenzhen greenway network, Activity distribution

## Abstract

This data presents the distribution of physical activities in the Shenzhen greenway network (GN) in January, April and July, 2014. The volunteered geographic on physical activity is overlaid with the greenways data, to describe the distribution of physical activities in the greenways. The data are summarized to show the distribution characteristics geographically from different aspects in Shenzhen, China. Data were used to explore the effect of the Shenzhen GN on supporting physical activities, “Where do networks really work? The effects of Shenzhen greenway network on supporting physical activities” (Liu et al., 2016) [2].

**Specifications Table**

**Table:** 

Subject area	Urban and landscape planning, Environmental behaviour
More specific subject area	Greenways, physical activity
Type of data	Table, text file, figures
How data was acquired	Geographic information system (GIS), Volunteered geographic information (VGI)
Data format	Raw and analyzed
Experimental factors	Physical activity VGI was recorded by a self-tracking applications and collected from social network, with workout routes and activity attributions. The VGI in January, April and July 2014 located in Shenzhen was selected to cover physical activities in different seasons.
Experimental features	The physical activity data and the Shenzhen greenway network (GN) data are overlaid and analyzed in ArcGIS 10, to figure out the distribution of activities in the Shenzhen greenways. The data were summarized in the administrative division.
Data source location	Shenzhen, China, Asia
Data accessibility	Data are included in this paper

**Value of the data**

• The data show the overall distribution of physical activities in the Shenzhen greenway network (GN), including the activities׳ presence, intensity and diversity, and can help researchers and practice professionals to evaluate the Shenzhen greenway utilization, find the popular segments and weakly-used part of the GN with ease in city-scale.
• The statistics of physical activities in each administrative and different greenway hierarchy can be used for further comparison and discussion on the greenway use from different aspects.
• The overall description of the greenway use can be discussed to reassess and improve the Shenzhen GN planning for physical activity promotion.

## 1 Data

The data fully utilize the physical activity Volunteered Geographic Information (VGI) by a popular self-tracking application from a social media, and intersect the VGI with the Shenzhen GN by GIS, to show the presence, intensity and diversity of the physical activities in the GN [2]. Shenzhen is made up of six districts, and the data are summarized in each administrative division, to show the spatial disparity of the greenway use.

## 2 Experimental design, materials and methods

### 2.1 Brief introduction of data resource and processing methods

#### 2.1.1 Data resource

The physical activity VGI is collected from Codoon, one of the most popular self-tracking applications in China. With Codoon, people can track the workout routes, record activity information such as activity date, types (classified as walking, jogging and cycling), distance, speed and duration, and upload them on Sina Weibo (one of the largest Chinese social networks). To cover the physical activities in different seasons, the physical activities VGI in January, April and July 2014 located in Shenzhen was selected from Sina Weibo. The records without routes and for activities shorter than 5 min were excluded.

**Fig. 1:**
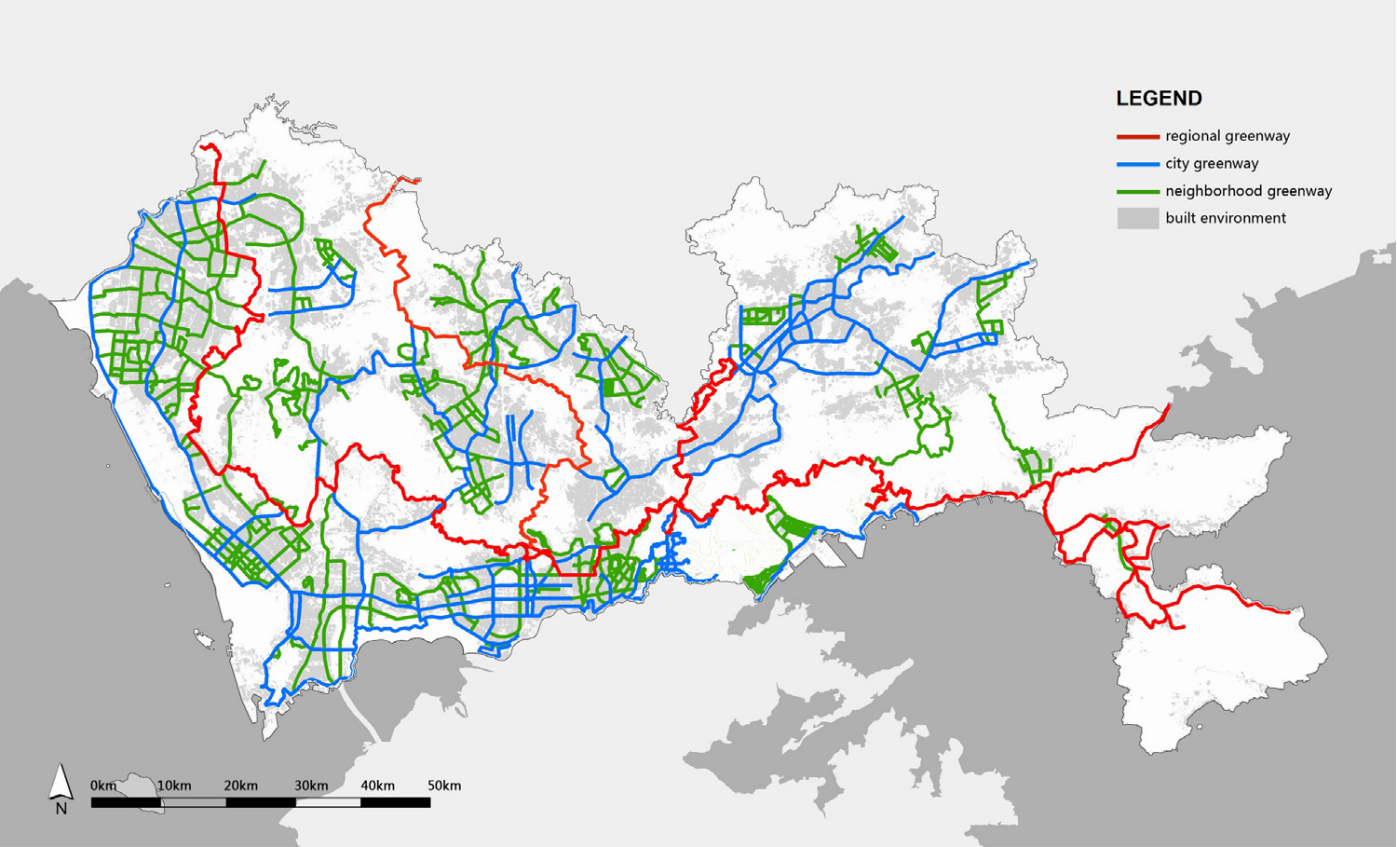
Shenzhen GN Map (2013). Original source: Shenzhen Greenways Map [4].

**Fig. 2:**
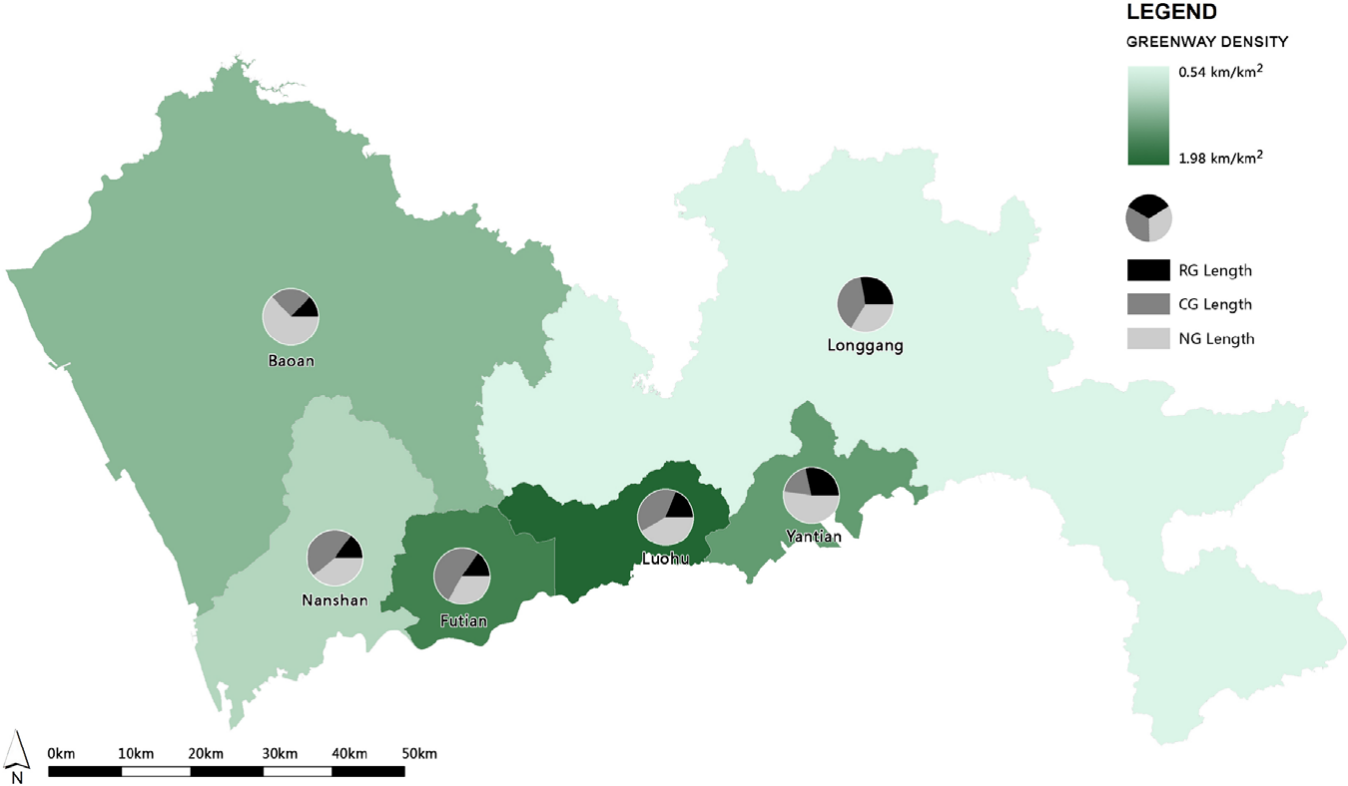
The greenway density per district; RG: Regional greenway, CG: City greenway, NG: Neighborhood greenway.

The Shenzhen GN data are from an official Shenzhen Greenway Map [4], in which the route and hierarchy of each greenway are mapped out (Fig. 1). Greenway density in the Shenzhen GN is the total length of the greenways per unit area, and was calculated and presented on the map by district (Table 1 and Fig. 2).

#### 2.1.2 Data processing

The physical activity data and the Shenzhen GN data were geocoded and overlaid in ArcGIS 10 with the Shenzhen local coordinate system to form the database. The distribution of physical activities in GN and related characteristics were calculated and shown with Arc toolbox and guided by NEAT-GIS Protocols [1].

### 2.2 The distribution of physical activities in GN

**Fig. 3:**
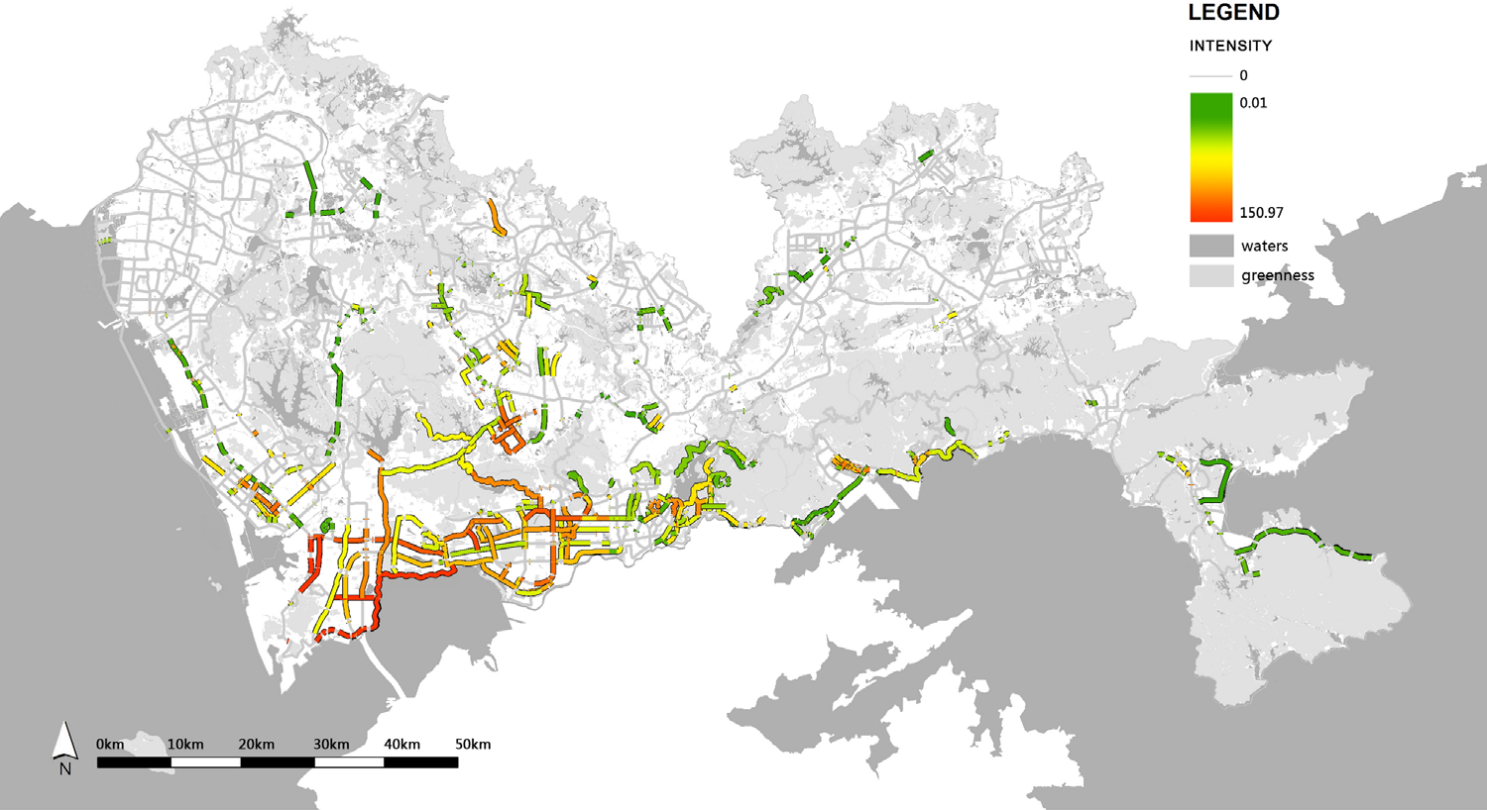
The distribution and the intensity of physical activities in the Shenzhen GN.

The physical activity VGI was overlaid on the GN data by means of an intersect tool, to show the distribution of physical activities in GN, in which the intersecting parts between the activity routes and the GN were considered to be the greenway sections with physical activities. By this method, the Shenzhen GN was divided into 636 greenway sections, of which 278 were with physical activities. The total distances of physical activities of each greenway section were divided by the length of the section in GIS, to present the distribution of the activity intensity within the GN (Fig. 3).

### 2.3 The proportion of the greenways in use by district

**Fig. 4:**
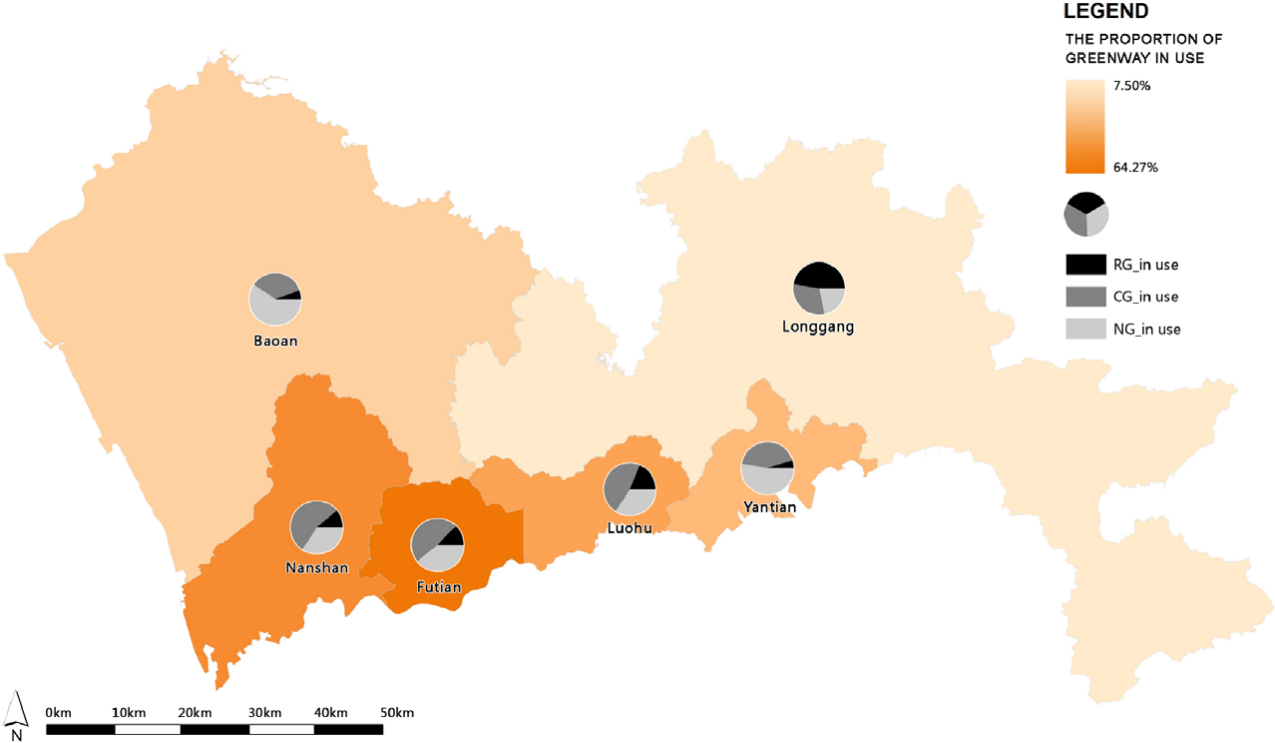
The proportion of the greenways in use per district; RG: Regional greenway, CG: City greenway, NG: Neighborhood greenway.

The proportion of the greenways in use is equal to the total length of greenway sections with physical activities divided by the total length of GN. The total length of greenway sections with and without physical activities were separately calculated by greenway hierarchy and by district. The average proportion of the greenways in use and the proportion of each hierarchy of greenways in use were then summarized and shown in each district (Table 2 and Fig. 4).

### 2.4 The intensity of physical activities by district

**Fig. 5:**
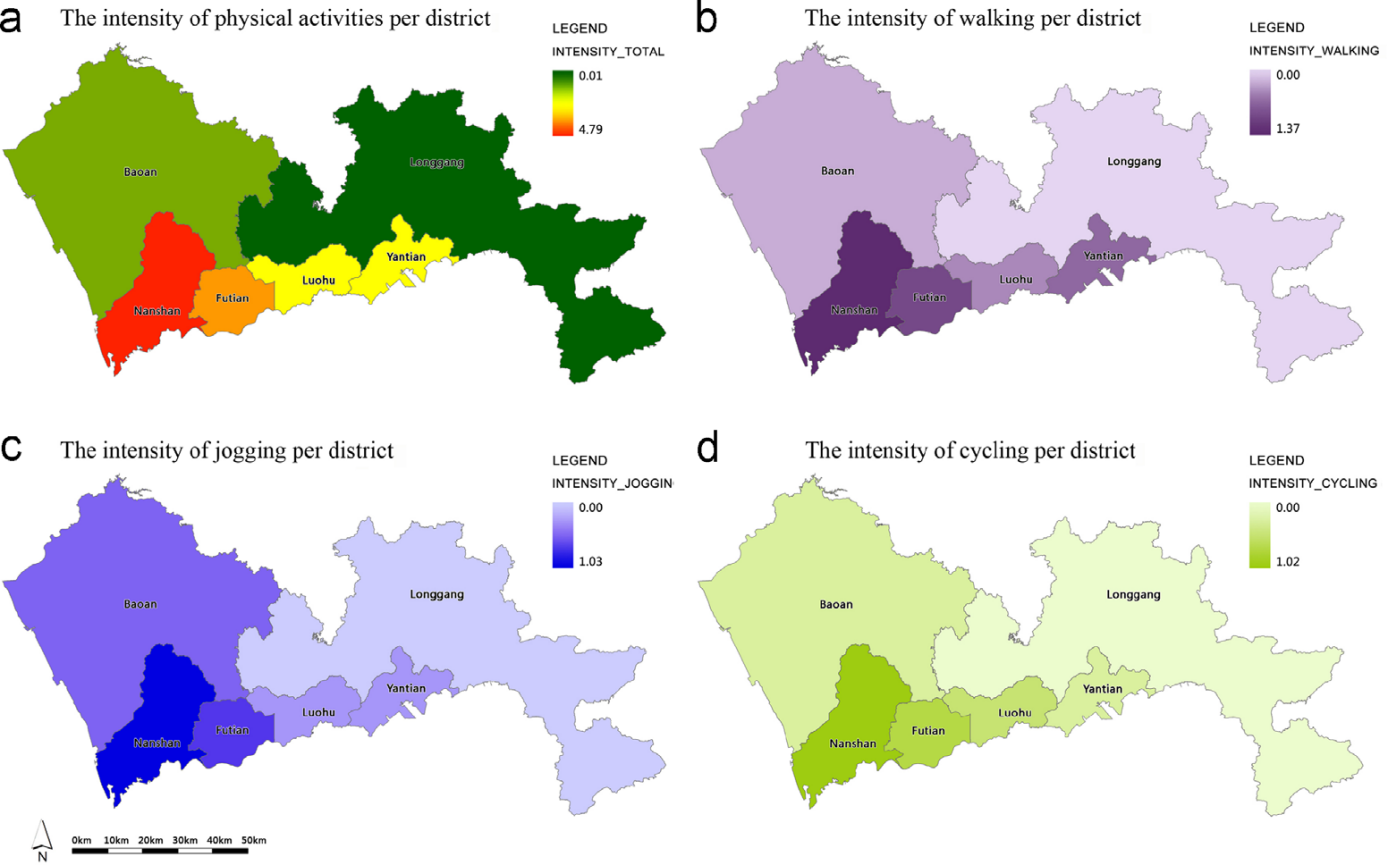
The intensity of physical activities in the Shenzhen GN per district.

The intensity of physical activity equals to the total workout distance of physical activities divided by the total length of the greenways in use. The total workout distances of physical activities within GN were added up by activity type and by district. Then the average physical activity intensity and each type of activity intensity of the greenways in use were available in each district (Table 3 and Fig. 5).

### 2.5 The diversity of physical activities by district

To show the physical activity diversity within GN, the entropy index [3] was introduced to measure the number of different physical activities in each greenway segment in use and the degree to which they were represented. The index varied between 0 and 1 (0 for maximum specialization, 1 for maximum diversification).

$$\mathrm{DIVERSITY}\_\mathrm{PA}=\{-\sum_{K}[\left(p_{i}\right)\left(\ln p_{i}\right)]\}/(\ln k)$$

where

*k*=Number of physical activity types (*k*=3, including walking, jogging and cycling)
pi=Distance proportion of each of the three physical activity types

**Fig. 6:**
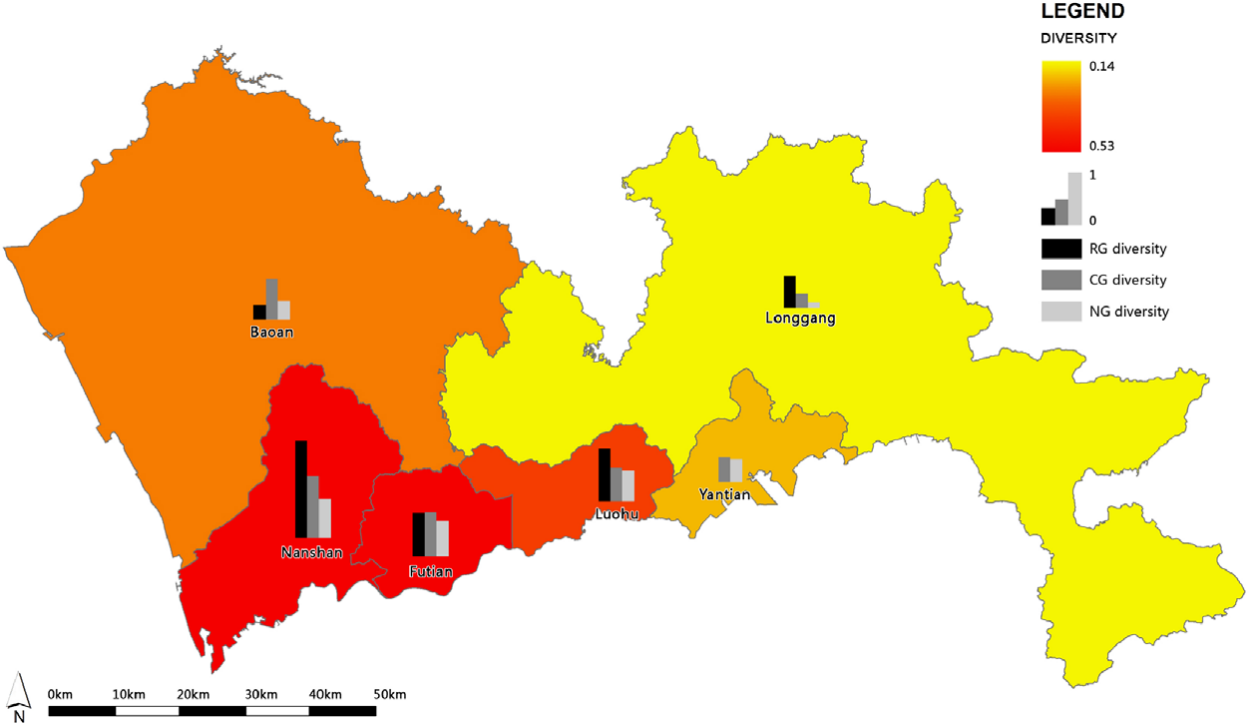
The diversity of physical activities in the Shenzhen GN per district; RG: Regional greenway, CG: city greenway, NG: Neighborhood greenway.

The physical activity diversity was calculated by hierarchy and by district, and the data were shown in Table 4 and Fig. 6.

## Figures and Tables

**Table 1:** The total length and the density of greenways by district.

District	District area (km^2^)	Greenway length (km)				Greenway density (km/km^2^)
		Regional greenway	City greenway	Neighborhood greenway	Total	
Baoan	721.19	87.39	162.46	425.58	675.43	0.94
Futian	78.66	17.26	56.98	37.34	111.59	1.42
Longgang	849.60	129.51	175.23	155.13	459.87	0.54
Luohu	78.75	29.45	61.21	65.16	155.82	1.98
Nanshan	179.14	20.56	63.71	53.82	138.09	0.77
Yantian	74.58	27.78	18.89	50.76	97.43	1.31
Total	1981.94	311.95	538.48	787.79	1638.22	0.82

**Table 2:** The presence of physical activities in the Shenzhen GN by district.

District	The length of greenways with activities (km)				The length of greenways without activities (km)				The proportion of greenways in use (%)
	Regional greenway	City greenway	Neighborhood greenway	Total	Regional greenway	City greenway	Neighborhood greenway	Total	
Baoan	5.17	30.63	52.94	88.74	82.22	131.83	372.64	586.69	13.14
Futian	9.06	34.60	28.04	71.70	8.20	22.36	9.30	39.86	64.27
Longgang	22.29	14.45	10.20	46.94	107.22	161.28	144.93	460.43	10.20
Luohu	12.65	32.40	23.24	68.29	16.80	28.81	41.92	87.53	43.82
Nanshan	9.13	45.64	28.36	83.13	11.43	18.07	25.46	54.96	60.19
Yantian	1.31	11.87	14.60	27.78	26.47	7.02	36.16	69.65	28.51
Total	59.61	169.59	157.38	386.58	252.34	369.37	630.41	1252.12	23.60

**Table 3:** The total length and the intensity of physical activities within the greenway sections in use by district.

District	The length of physical activities (km)				Mean value of physical activity intensity			
	Walking	Jogging	Cycling	Total	Walking intensity	Jogging intensity	Cycling intensity	Overall intensity
Baoan	9.81	85.36	9.86	105.03	0.01	0.13	0.01	0.16
Futian	42.66	129.92	12.09	184.67	0.38	1.16	0.11	1.66
Longgang	0.18	3.75	0.69	4.62	0.00	0.01	0.00	0.01
Luohu	10.02	18.11	3.39	31.52	0.06	0.12	0.02	0.20
Nanshan	189.33	330.13	142.08	661.54	1.37	2.39	1.03	4.79
Yantian	7.00	11.41	0.88	19.29	0.07	0.12	0.01	0.20
Total	259	578.68	168.99	1006.67	0.14	0.32	0.09	0.56

**Table 4:** The diversity of physical activities within the greenway sections in use by district.

District	Mean value of physical activity diversity of the greenways in use			
	Regional greenway	City greenway	Neighborhood greenway	Overall GN
Baoan	0.15	0.41	0.19	0.22
Futian	0.44	0.45	0.36	0.40
Longgang	0.32	0.15	0.06	0.14
Luohu	0.54	0.34	0.31	0.33
Nanshan	0.97	0.62	0.39	0.53
Yantian	0.00	0.25	0.23	0.23
Total	0.35	0.35	0.22	0.27
