# Supplementary material for: Data on the distribution of physical activities in the Shenzhen greenway network with volunteered geographic information
Source: Data Brief. 2016 May 12;8:14–20. doi: 10.1016/j.dib.2016.05.006 (PMC4878787; doi:10.1016/j.dib.2016.05.006)
Supplement: Supplementary file 1 — Supplementary material [file mmc1.docx]

Conflict of interest: None

*Data in Brief*

**Title:** Data about the distribution of physical activities in the Shenzhen greenway network with volunteered geographic information

**Authors:** Kun LIU^1, 2^, Kin Wai Michael SIU^1,^*, Yong Xi GONG^2^, Yuan GAO^2^, Dan LU^2^

**Affiliations:** 1. The Hong Kong Polytechnic University, School of Design, Hong Kong

2. Harbin Institute of Technology, Shenzhen Graduate School, Shenzhen, China

* Corresponding author

**Contact email:** m.siu@polyu.edu.hk

Signed by


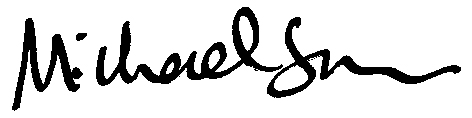


Kin Wai Michael Siu

Corresponding author

15 April 2016
